# SEMAPHORINS and their receptors: focus on the crosstalk between melanoma and hypoxia

**DOI:** 10.1186/s13046-021-01929-3

**Published:** 2021-04-15

**Authors:** Elisabetta Valentini, Marta Di Martile, Donatella Del Bufalo, Simona D’Aguanno

**Affiliations:** Preclinical Models and New Therapeutic Agents Unit, IRCCS Regina Elena National Cancer Institute, Rome, Via Chianesi 53 (00144), Rome, Italy

**Keywords:** Hypoxia, Semaphorins, Plexins, Neuropilins, Melanoma, Cancer

## Abstract

Hypoxia, a condition of oxygen deprivation, is considered a hallmark of tumor microenvironment regulating several pathways and promoting cancer progression and resistance to therapy. Semaphorins, a family of about 20 secreted, transmembrane and GPI-linked glycoproteins, and their cognate receptors (plexins and neuropilins) play a pivotal role in the crosstalk between cancer and stromal cells present in the tumor microenvironment. Many studies reported that some semaphorins are involved in the development of a permissive tumor niche, guiding cell-cell communication and, consequently, the development and progression, as well as the response to therapy, of different cancer histotypes, including melanoma.

In this review we will summarize the state of art of semaphorins regulation by hypoxic condition in cancer with different origin. We will also describe evidence about the ability of semaphorins to affect the expression and activity of transcription factors activated by hypoxia, such as hypoxia-inducible factor-1. Finally, we will focus our attention on findings reporting the role of semaphorins in melanocytes transformation, melanoma progression and response to therapy. Further studies are necessary to understand the mechanisms through which semaphorins induce their effect and to shed light on the possibility to use semaphorins or their cognate receptors as prognostic markers and/or therapeutic targets in melanoma or other malignancies.

## Background

Semaphorins were originally identified in the nervous system for their ability to regulate axon guidance through the interaction with their receptors, plexins and neuropilins [1]. They are also involved in cardiac and skeletal development [2], regulation of immune system [3] and pathways such as apoptosis [4], autophagy [5] and senescence [6]. Several human diseases including immunopathology [7], neurodegenerative [8] and cardiovascular [9] diseases show aberrant expression of semaphorins.

Semaphorins also show a pleiotropic role in cancer etiology. In particular, through the crosstalk between cancer cells and stromal cells present in tumor microenvironment (i.e. fibroblasts, immune and endothelial cells), semaphorins and their cognate receptors are involved in various aspects of tumor development and progression, sometimes inducing antagonist effects*.* For instance, while several semaphorins have been reported to reduce tumor growth and angiogenesis, others are found to promote tumor growth or metastasis [10]. The ability to induce vessel normalization, a phenomenon acting to re-establish normal vessels/network in terms of structure and function [11], has been also reported for some semaphorins [12–14]*.* The involvement of semaphorins/receptors axis in the response to therapy, and their role as potential therapeutic targets in cancer has been also evidenced [15–17]*.* Moreover, semaphorins and their cognate receptors are involved in controlling cancer stem cell phenotype, contributing to tumor progression, resistance to therapies, and metastasis initiation [18].

Hypoxia (low oxygen concentration) and nutrient deprivation, caused by inappropriate blood supply, represent important hallmarks of tumor microenvironment. Semaphorins/semaphorin receptors are considered relevant drivers of angiogenesis and regulators of tumor progression and response to therapy, as well as cellular metabolism and genomic stability [19]. In response to hypoxia, tumors activate a general adaptive response allowing their growth under unfavourable conditions.

Hypoxia-inducible factors (HIFs) are responsible for the transcriptional responses to hypoxic stress. These transcription factors are composed of an alpha subunit (HIF-1α, HIF-2α and HIF-3α), and a beta subunit (HIF-1β) [20]. While mRNA of alfa subunits (HIFs- α) are not altered by exposure to hypoxia, alfa, but not beta, proteins are stabilized by hypoxia. As consequence, HIFs-α translocated in the nucleus and dimerize with HIF-1β, activating the transcription of hundreds target genes by binding to hypoxia response elements (HRE) located in either the promoter or enhancer regions, finally resulting in tumor cellular adaptation to hypoxia [21–23]. The consensus core of HRE sequence is 5′-RCGTG-3′ (where R is A or G).

In the last years a huge amount of data has been obtained shedding novel insights on the mechanisms through which hypoxia affects tumor progression and response to therapy. Semaphorins and their receptors represent a pathway frequently regulated by hypoxia and the consequence of this regulation has been often characterized in different type of cancers, including melanoma.

Melanoma is a neoplasia originating from the transformation of normal melanocytes, pigment-producing cells of epidermis. It rapidly metastasizes early in tumor progression. Despite the impressive results of recent targeted and immune therapies [24], metastatic melanoma is still considered an incurable disease. Melanoma microenvironment, and in particular hypoxic condition, regulates several cellular pathways and influences melanoma progression and its response to anticancer therapies [25, 26]. Tumor-stromal interaction is a relatively understood phenomenon deeply studied in the last years. Several efforts have been devoted to the understanding of functions that semaphorins and their receptors play in both melanoma cells and they precursors, melanocytes. Mediating several signaling cascades and playing an important role in tumor microenvironment, semaphorins and their cognate receptors show pivotal roles in melanoma initiation, pathobiology and response to therapy, and could represent a potential approach for melanoma and, more in general, for cancer therapy.

**Fig. 1:**
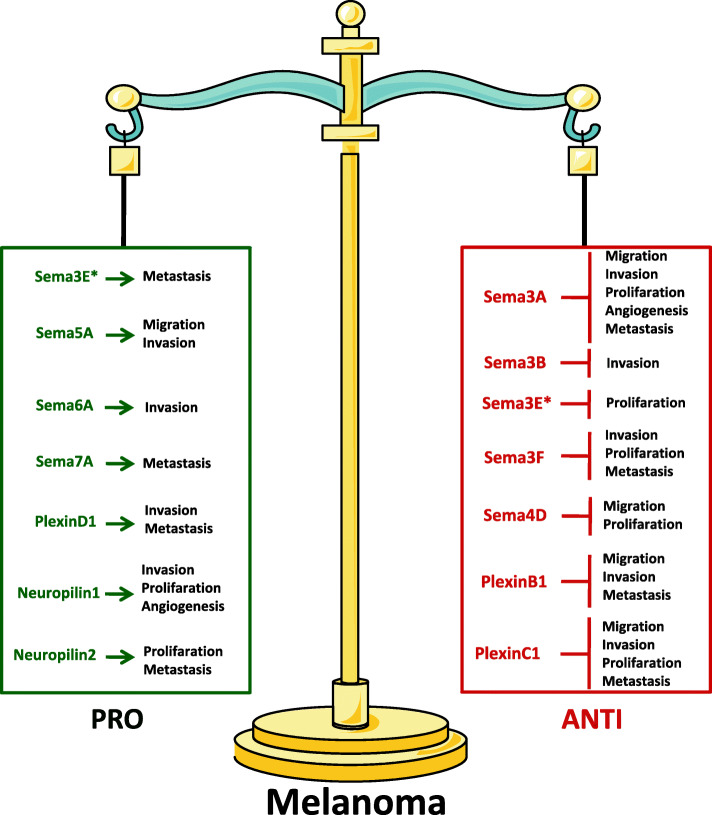
Schematic representation of the pro- and anti-tumoral role of semaphorins and their receptors (Neuropilin1–2, PlexinB1-C1) in melanoma progression. *Both pro-tumoral and anti-tumoral effects have been observed for Sema3E

In this review we will focus on the role played by semaphorins and their receptors in melanoma pathobiology. Figure 1 shows a schematic representation of pro- and anti- tumoral role of semaphorins and their receptors in melanoma progression. As several reviews discussed the involvement of semaphorins/semaphorin receptors axis in component of tumor microenvironment, such as fibroblasts, endothelial and immune cell, [10, 27–29] this issue is out of the scope of this review.

### Biochemical aspects of Semaphorins and their receptors

**Fig. 2:**
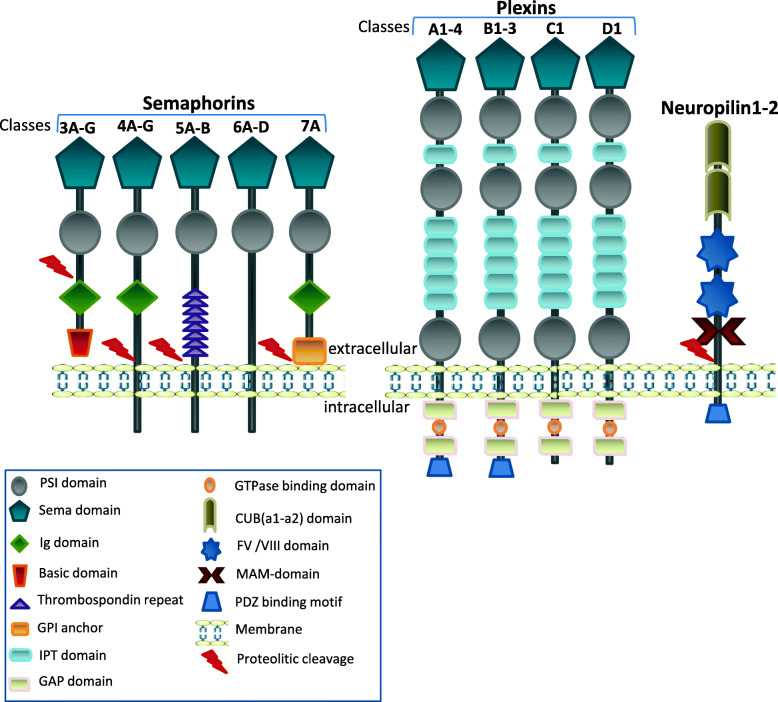
Schematic representation of the protein structure of semaphorins and their receptors. Plexin-Semaphorin-Integrin (PSI); Semaphorin (SEMA); Immunoglobulin-like (Ig); Transcription factors (IPT); GTPase-Activator Protein (GAP); Complement C1r/C1s, Uegf, Bmp1 (CUB); Coagulation factors V and VIII (FV/FVIII); Meprin, A5 protein, receptor protein tyrosine phosphatase Mu (MAM); PSD95/Dlg/ZO-1 (PDZ)

The members of semaphorin family are more than 20 secreted, transmembrane and GPI-linked glycoproteins. By a structural point of view these proteins show a highly conserved N-terminal sema domain of about 500 amino acids, a plexin–semaphorin–integrin domain and distinct protein domains that further define semaphorins, including immunoglobulin-like, thrombospondin, and basic C-terminal domains [30] (Fig. 2). Semaphorins are grouped into 8 classes: class 1 and 2 are found in invertebrates, class 3–7 are typical of vertebrates, and class 8 is viral-encoded. Class 1 and class 4–7 semaphorins are mainly membrane-associated, whereas class 2, 3, and 8 are mostly secreted. Membrane-associated semaphorins are able to mediate direct cell–cell interactions, while secreted semaphorins, are involved in chemoattraction/chemorepulsion activity.

All these functions are mediated by the binding with specific receptors. Two groups of proteins, plexins and neuropilins, have been identified as main semaphorin receptors [31]. Different semaphorins are able to activate specific plexins directly or through the interaction with the coreceptor neuropilin (Tables 1 and 2). There are 9 plexins in vertebrates, subdivided into 4 families: plexinA (1–4), plexinB (1–3), plexinC1 and plexinD1 (Fig. 2). Plexins are composed of an extracellular portion typically formed by 10 domains, including a sema domain, which differs from that of semaphorins for the lack of ability to dimerize, a membrane-spanning region and a cytoplasmic segment, which interacts with intracellular signalling molecules [32]. A shorter intracellular domain, containing a PSD95/Dlg/ZO-1 (PDZ) binding motif, characterizes the other class of receptors, composed of neuropilins − 1 and − 2, [33]. In the extracellular segment of neuropilins, there are different domains and among these there is also the binding site for vascular endothelial growth factor (VEGF), hepatocyte growth factor (HGF), fibroblast growth factor-2, platelet-derived growth factor-beta, transforming growth factor-beta and other ligands [34, 35]. Neuropilins interaction with plexins facilitates the transduction of pro-angiogenic signals [36]*.*

**Table 1:** Semaphorins which activate plexins directly

SEMA	PlexinA1	PlexinA2	PlexinA3	PlexinA4	PlexinB1	PlexinB2	PlexinB3	PlexinC1	PlexinD1
Sema3E									**x**
Sema4A					**x**	**x**	**x**		**x**
Sema4C						**x**			
Sema4D					**x**	**x**			
Sema5A	**x**		**x**				**x**		
Sema5B	**x**		**x**						
Sema6A		**x**		**x**					
Sema6B		**x**		**x**					
Sema6D	**x**								
Sema7A								**x**

**Table 2:** Semaphorins which activate plexins via neuropilins

Plexin(A1–4)-NRP1	Plexin(A1–4)-NRP2	PlexinD1-NRP1-NRP2
Sema3A	Sema3B	Sema3C
Sema3B	Sema3C	
Sema3C	Sema3D	
Sema4A	Sema3G	
Sema3D	Sema3F	

### Regulation of SEMAPHORINS in cancer cells by hypoxic condition

**Fig. 3:**
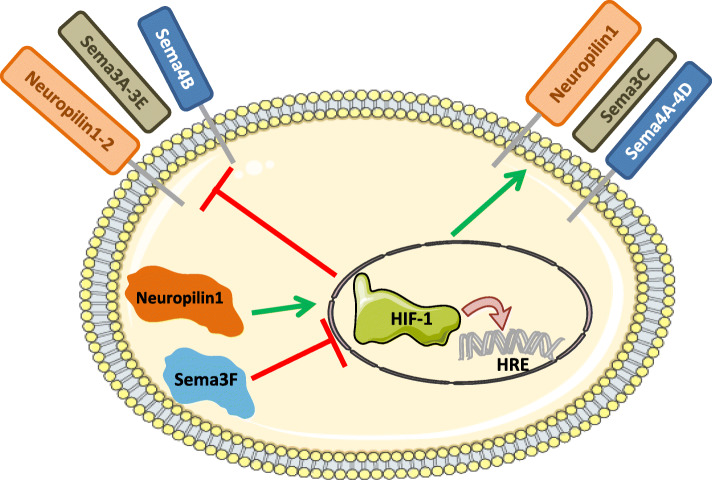
Schematic representation of the crosstalk between hypoxia-inducible factor 1 (HIF-1) and semaphorins or their receptors in tumors. HIF-1 through the binding to the hypoxia responsive element (HRE) present in the promoter of semaphorin or neuropilin genes induces (green arrow) or blocks (red line) their expression. Inhibition of HIF-1 expression by Sema3F (red line) and induction of HIF-1 expression by neuropilin1 (green arrow) are also reported

Semaphorins have been shown to have a pivotal role in the signal transduction, microenvironment regulation and cell-cell communication, thanks to the binding with their cognate receptors. A crosstalk between hypoxia and semaphorins expressed by different cancer models has been largely described (Fig. 3).

### Semaphorin3A (Sema3A), Semaphorin3B (Sema3B), Semaphorin3C (Sema3C), Semaphorin3E (Sema3E)

In prostate cancer cells, HRE sequences have been found in the promoter of Sema3A, Sema3B, Sema3C, Sema3E, and Sema3F genes, but not in Sema3D*.* The authors reported up-regulation of Sema3C and downregulation of Sema3A and Sema3E levels after exposure to hypoxia or hypoxic mimetic agents*,* thus suggesting a different role of Sema3 family members in the progression of prostate cancer [37].

By using mice models of both pancreatic neuroendocrine and cervical carcinoma, it has been demonstrated the ability of Sema3A to counteract the activation of HIF-1α and several hypoxia-dependent signalling pathways induced by sunitinib, an anti-angiogenic drug, with a consequent improvement of tumor tissue oxygenation. The authors suggested re-expression of Sema3A as a possibility to inhibit the metastatic dissemination induced by anti-angiogenic treatment [38].

The group of Casazza demonstrated that hypoxia-induced Sema3A in lung carcinoma cells is responsible for the entry of tumor-associated macrophages into hypoxic niches through neuropilin1-mediated signalling, thus indicating the possibility to modulate macrophage localization and phenotype to drive them against cancer [39, 40].

### Semaphorin3F (Sema3F)

Sema3F has been reported to affect several signalling pathways in cancer cells from different histotypes [41]. Among these, the negative regulation of HIF-1α/VEGF axis, tumor growth and microvessel density in lung cancer has been reported when Sema3F was overexpressed [42]*.* In agreement with these results, Sema3F loss in lung cancer cells was associated with increase of HIF-1α protein under hypoxic condition, thus supporting the relevance of Sema3F in the progression of lung cancer and its role as anti-angiogenic protein [43]*.* Sema3F interacts with neuropilin1, but it has higher affinity for neuropilin2, promoting its tumor-suppressive activity in many cancer models [44]. This interaction with neuropilin2 is also involved in its anti-lymphangiogenic function in vivo, showing a direct chemorepulsive effect on lymphatic endothelial cells [45, 46]. However, further experiments will be needed to clarify the anti-lymphangiogenic role of Sema3F.

### Semaphorin4A (Sema4A)

Chromatin immunoprecipitation assay evidenced the ability of HIF-1α, but not HIF-2α, to bind the promoter of Sema4A gene, in cooperation with hypoxia in breast cancer cells. Moreover, silencing of Sema4A under hypoxic condition was able to reduce hypoxic effects, such as induction of VEGF and activation of MAPK, AKT and STAT3 and to induce apoptosis*.* As Sema4A has been reported to be involved in the progression of breast cancer and its expression was higher in the tissues and serum of patients when compared with normal tissues [47], these results could promote the use of Sema4A as a potential target for treatment of breast carcinoma.

### Semaphorin4B (Sema4B)

The group of Jian H demonstrated the role of Sema4B in suppressing the growth and the metastatization of non-small cell lung cancer [48, 49], shedding light on the ability of hypoxia to regulate invasion of lung carcinoma through transcriptional repression of Sema4B mediated by the binding of HIF-1α to HRE present in Sema4B gene. Sema4B overexpression also reduced hypoxia-induced invasion of lung cancer cells [50]*.*

### Semaphorin4D (Sema4D)

Sema4D is induced by hypoxia through a HIF-1α-dependent mechanism in tumors from different origin such as colon [51, 52]*,* oral squamous cell [53, 54] and lung [55] carcinoma, with consequent regulation of tumor vascularization. Several HRE within Sema4D promoter have been identified [53, 56–58]. Immunohistochemical analysis evidenced the expression of both Sema4D and HIF-1α in about 60% of colorectal carcinoma tissue and in about 10% of normal mucosa. It also demonstrated a positive correlation between Sema4D and HIF-1α, as well as a correlation of both HIF-1α and Sema4D with metastatization and TNM (tumor-node-metastasis) stage, and their role as prognostic factor in patients with colorectal carcinoma [51, 52].

Similarly, the ability of Sema4D to induce tumor growth and vascularization through a HIF-1-dependent mechanism was demonstrated, together with a correlation between the expression of Sema4D and the activity of HIF-1 in specimens from head and neck squamous carcinoma [53, 54] and from epithelial ovarian cancer [59].

Induction of Sema4D by hypoxia through a HIF-1α-dependent mechanism has been also evidenced in lung cancer, and up-regulation of a disintegrin and ADAM17 expressions by hypoxia has been identified as the cause of Sema4D induction [55]*.*

By screening the 5′ non-coding region before the ATG start codon of Sema4D in endothelial and cancer cells including colorectal, nasopharyngeal and ovarian carcinoma, and leukemic cells, a recent paper identified four possible HRE, with different functions depending on the cell type. Two mutations (T471C and C862T) close to the HIF-1 binding site were particularly expressed in cancer cells and responsible of change in the gene structure and in the activation of target genes [56]*.* These data could be useful for the identification of new therapeutic options.

### Regulation of NEUROPILINS in cancer cells by hypoxic condition

#### Neuropilin1

The expression of neuropilin1 is induced by hypoxia in several normal cell types, such as endothelial cells [60] and embryonic stem cells [61]*.* On the contrary, neuropilin1 has been identified as a gene repressed by HIF-1α in complex with E2F7 and regulating the spinal motoneurons axon guidance in zebrafish embryos [62]*.*

Scattered evidence is furnished regarding the effect of hypoxia on neuropilin1 expression in cancer cells. In particular, hypoxia and nutrient deprivation induced degradation of neuropilin1, but not neuropilin2, in both breast and prostate carcinoma cells [5]. Hypoxia also decreased neuropilin1 expression in malignant astrocytoma [63]. On the contrary, hypoxic condition increased the expression of neuropilin1 in human neuroblastoma [64] and pancreatic cancer [65] cell lines. An association between HIF-1α and neuropilin1 in pancreatic cancer tissues has been reported, together with a positive regulation of HIF-1α by neuropilin1, suggesting a feedback loop between HIF-1α and neuropilin1, but the authors did not investigate the mechanism of this regulation [65]. Surprisingly, contrary to the study of Bae et al, demonstrating degradation of neuropilin1 by hypoxia in breast cancer cells [5], the findings from Barr et al [66] evidenced induction of neuropili1 expression after exposure to hypoxia. This discrepancy could be related to the different cell context used.

A positive correlation between the intratumoral gene expression of neuroplin1, HIF-1α, HIF-2α, VEGF, VEGF receptor-1, lactate dehydrogenase and glucose transporter-1 was evidenced in tissues samples from metastatic colorectal cancer patients [67]*.*

Up-regulation of neuropilin1 by a hypoxia-mediated HIF-1α-dependent mechanism has been reported to play a critical role in the in vivo vasculogenic mimicry, as well as tumor formation and growth of fibrosarcoma [68].

By using human cervical cancer cells and tumor specimens, neuropilin1 has been found increased by hypoxia and was described as a key mediator in hypoxic cancer cells for the macrophage recruitment and polarization into the pro-tumoral M2 phenotype, thus providing a new insight into the mechanisms through which neuropilin1 acts in response to hypoxia microenvironment [69]*.*

Findings from Zhuang group, obtained by using human specimens from hepatocellular carcinoma patients and mouse xenograft models, demonstrated that peritumoral hypoxia was negatively correlated with peritumoral neuropilin1 and VEGF receptor-2 (VEGFR-2) expression, and with tumoral/peritumoral microvascular density. More importantly, the authors evidenced that high expression of neuropilin1 and VEGFR-2 by peritumoral liver cells predict a favourable postoperative outcome of hepatocellular carcinoma patients with increased time to recurrence and overall survival [70]. These results are consistent with those obtained in acute lymphoblastic leukaemia and acute myeloid leukaemia [71]*,* but contrasting with those reported in bladder cancer patients [72] demonstrating, respectively, neuropilin1 association with favourable or adverse prognosis.

To corroborate the cancer specific effect of neuropilin1, a previous study performed on patients with distant relapse and without relapse after radical prostatectomy reported no significant difference for HIF-1α and neuropilin1 expression in tumor cells [73]*.* In agreement, there is evidence that neuropilin1 either reduces tumor growth and spread in human pancreatic adenocarcinoma cells [74] or facilitates tumor growth and progression in lung and colon cancer [75–77], or protect from hypoxia induced apoptosis in breast cancer cells [78]*.* These data supported the idea that neuropilin1 effect is closely related with tumor histotypes.

Neuropilin1 has been also reported to play a critical role in the ability of circular HIPK3 (circHIPK3) to promote metastasis in gastric cancer under long-term exposure to hypoxia. In particular, under hypoxia circHIPK3 is induced by HIF-2α and, through the direct interaction and sponging with miR-653-5p and miR-338-3p, abrogated the suppression of neuropilin1 with consequent promotion of metastases. In support of these results, dataset analysis indicated neuropilin1 as a poor prognostic biomarker for patients with gastric cancer [79]*.*

#### Neuropilin2

Hypoxia or hypoxia mimetic agents have been reported to negatively control the expression of neuropilin2 at the transcriptional level in hepatoma, glioblastoma and melanoma models [80, 81] . Further characterization in glioblastoma reported neuropilin2 repression by hypoxia through the HIF1-α, but not HIF2-α involvement, with consequent inhibition of the antitumorigenic activity of Sema3F and enhancement of VEGF pro-angiogenic activity [80]*.* Exposure to hypoxia of colon cancer cells with reduced levels of neuropilin2 induced an increase of apoptotic cells [82]. These results shed light on the useful of neuropilin2 as therapeutic target in the treatment of glioblastoma, melanoma, colon cancer, as well as hepatoma.

As regarding the other class of semaphorins receptors, the plexins, no data about their regulation by hypoxia are available.

### Involvement of SEMAPHORINS in melanoma progression and response to therapy

**Fig. 4:**
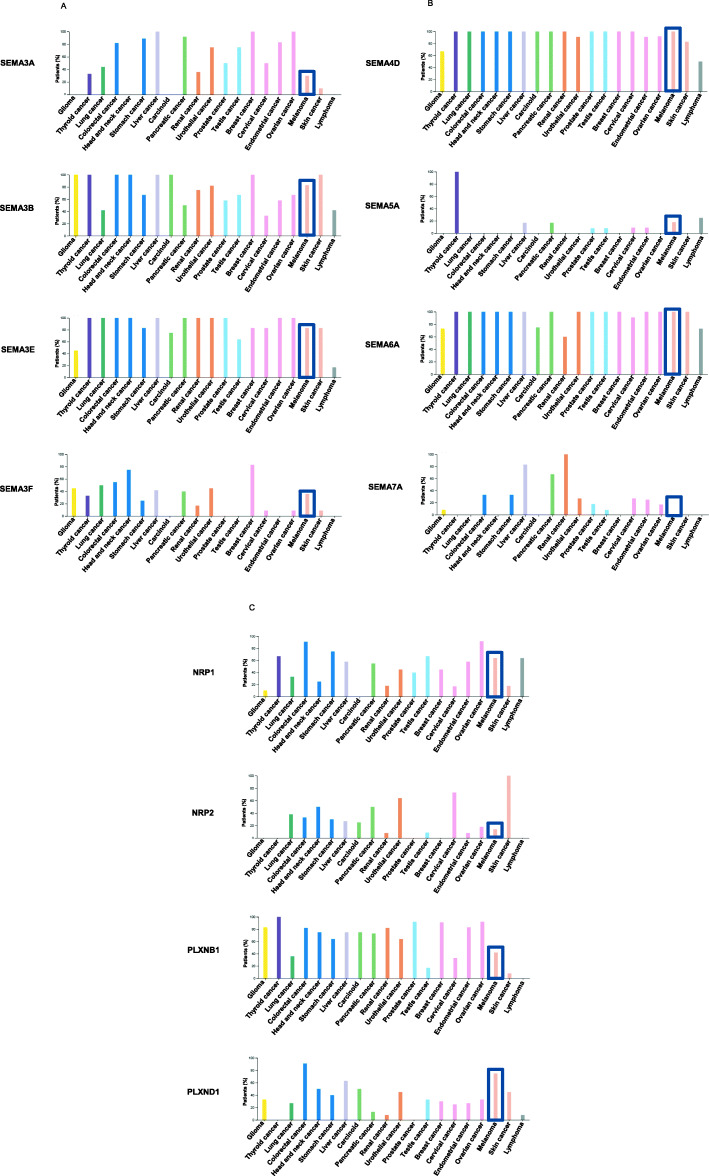
Protein expression of selected semaphorins and their receptor in cancer. Bar charts showing the protein expression in different tumor histotypes of (**a**) Sema3A, 3B, 3E, 3F, (**b**) Sema4D, 5A, 6A, 7A and (**c**) their cognate receptors neuropilin1, 2 (NRP1, 2) and plexinB1, D1 (PLXNB1, D1) detected by immunohistochemistry and reported as percentage of positive patient samples. Data are from The Human Protein Atlas database (https://www.proteinatlas.org/). We reported the percentage of positive biopsies from melanoma and other tumor histotypes for those semaphorins and their cognate receptors that have been described to play a role in melanoma progression

Interrogating the Protein Atlas Database (https://www.proteinatlas.org/) we found that semaphorins and their cognate receptors are all expressed at RNA transcript level in a wide range of tumor histotypes, including melanoma. At protein level, several semaphorins, plexins and neuropilins have been detected in tissue cancer available in the database, including melanoma, except for Sema4A, Sema4F, Sema4G, Sema6B, plexinA4, plexinB3 for which melanoma specimens resulted weakly positive or prevalently negative. Information about the protein expression levels of Sema3C, Sema6D, Sema6C, plexinA3 and plexinC1 was not available due to pending tissue analysis. As displayed in Fig. 4, the percentage of positive biopsies of the selected semaphorins or their receptors in melanoma ranged from 20 to 100%. Of note, in agreement with finding demonstrating absence of detectable Sema7A protein in a panel of human melanoma cell lines [83], the analysed melanoma tissues resulted prevalently negative for this semaphorin.

#### Sema3A

The Sema3A/Neuropilin1 pathway has been found to drive the fate of neural crest cells, which are motile embryonic cells differentiating into specific cell types, including melanocytes [84].

Multiple in vitro approaches and in vivo mice models identified Sema3A as a tumor suppressor for melanoma. In particular, overexpression of Sema3A in melanoma models was able to suppress cell migration, invasion and proliferation, as well as to inhibit in vivo tumor growth, angiogenesis and metastasization. An enhanced response to dacarbazine and curcumin was also observed in melanoma cells expressing high levels of Sema3A [85]*.* In agreement with these findings, a recent study performed on 170 patients with cutaneous melanoma, evidenced reduced expression of Sema3A in intermediate and thick melanomas that was associated with metastasis and poor survival. The authors indicated Sema3A as a tumor suppressor gene and a valid prognostic biomarker for the subtype of patients with Breslow thickness > 1.0 mm [86]*.*

#### Sema3B

The only evidence about Sema3B role in melanoma was published in 2001 [87]*.* This study also represents the first publication about semaphorins in melanoma. In particular, comparative hybridization of cDNA arrays between highly and weakly invasive melanoma cells, and further confirmation by quantitative RT-PCR, identified Sema3B downregulation in highly invasive cells*.* To date no further results have been published about the expression or the role of this semaphorin in melanoma.

#### Sema3E

Contrasting results were obtained regarding the role of Sema3E in melanoma. In 2008 Sema3E was found abundantly expressed both in naevocellular and dysplastic naevi, while no detectable staining was observed in melanoma metastasis by Roodink et al., indicating an inverse correlation between Sema3E and tumor progression and suggesting for this molecule the role of tumor suppressor gene [88]. An inverse correlation between Sema3E and plexinD1 during tumor progression was also evidenced [88]. On the contrary, two years later, in agreement with data obtained with mammary adenocarcinoma [89], Casazza et al. reported a positive correlation between Sema3E and melanoma progression, being all the Clark levels III and IV metastatic melanoma specimens positive for the expression of Sema3E, while benign skin lesions showing only a low percentage of positivity. In addition, overexpression of Sema3E in a xenograft model of metastatic melanoma was reported by Casazza group to increase, rather than to inhibit, the metastatic spread as reported by Roodink group [88, 90]. In line with these results, Sema3E overexpression increased in vitro tumor progression-associated properties and its knockdown reduced metastatic ability in mouse models. Very surprisingly a paradoxical effect was observed by Casazza, being tumor growth inhibited in Sema3E overexpressing tumors, while metastases were induced. These results indicate that tumor growth is not necessarily correlated with tumor metastatization. The authors hypothesized Sema3E/plexinD1/ErbB2-Neu as a possible axis able to promote invasive behaviour [90]. On the contrary, Roodink et al. excluded Sema3E as an activating ligand for plexinD1 [88]. These contrasting results indicate that great attention and new studies are necessary before considering Sema3E or plexinD1 as possible target for melanoma treatment [91]*.*

#### Sema3F

Recently, the impact of Sema3F for patterning of neural crest-derived melanocytes has been evidenced in a model of lamprey through the use of CRISP/Cas technology [92]*.*

While a general consensus for Sema3F as a tumor suppressor in melanoma has been reported, contradictory results were obtained regarding the effect of Sema3F on in vitro proliferation of melanoma cells. In particular, while melanoma cell proliferation was not affected by Sema3F in results obtained by Bielenberg group [45], negative effect on cell proliferation was demonstrated by Chabbert-de Ponnat et al. either with the addition of exogenous soluble Sema3F, or after induced overexpression of the recombinant molecule [93]*.* The authors attributed the discrepancy to the different amount of Sema3F secreted by the different cells used in the two studies.

In support of its role as a tumor suppressor gene, Sema3F was found downregulated in highly metastatic human melanoma cell lines in vitro and in vivo*,* and cells overexpressing Sema3F showed a reduced cell adhesion and migration toward fibronectin and were chemorepulsive for vascular and lymphatic endothelial cells expressing neuropilin2, thus indicating the effect of Sema3F on stromal cells, other than melanoma cells [45]. Even if Sema3F overexpressing tumors obtained after injection of melanoma cells in mice, presented similar volume, they were less vascularized and showed features of less aggressive tumors when compared to parental ones. They also showed reduced lymph node and lung metastases [45, 94]*.*

Comas’ group reported that in high metastatic cells Sema3F expression is under the control of c-myc/Id2 pathway, and that Id2 enhanced migratory and invasive ability of melanoma cells through downregulation of Sema3F in a neuropilin2 dependent manner. The authors also suggested the development of inhibitors of c-myc/Id2 expression and/or activity to enhance Sema3F levels as a possible strategy to block metastasis [94]*.*

#### Sema4D

Although at low levels, Sema4D is expressed by melanocytes from the skin and by melanocytes cultured in vitro [95]. In these cells, Sema4D activated Erk1/Erk2 and stimulated proliferation in a plexinB1-dependent manner, and induced survival in response to ultraviolet irradiation, protecting melanocytes from ultraviolet induced apoptosis. Sema4D also suppressed the activation of c-Met tyrosine kinase receptor in response to the HGF in a plexinB1 dependent manner through Shp2, a non-receptor protein tyrosine phosphatase [95, 96]. c-Met is a factor regulating proliferation, migration and survival of melanocytes, mediating their transformation and melanoma progression [97, 98]*.*

In a recent work, Chen et al. analyzing the expression of different genes in human melanoma samples, have showed the association of Sema4D with both relapse-free survival and overall survival and lower Sema4D expression levels in melanoma tumors than normal skin. These data shed light on using Sema4D either as prognostic marker or as potential molecular target for the treatment of this neoplasia [99].

#### Sema5A

In agreement with previously published results obtained in gastric [100] and pancreatic [101] cancer, in 2018 we provided evidence supporting a positive role of Sema5A in melanoma migration and invasion, through Akt/ERK phosphorylation, and in the formation of vasculogenic structures. The relevance of Sema5A in melanoma progression was also furnished by public database of microarray profiling and by analysis of human melanoma specimens, indicating higher level of Sema5A in more aggressive melanoma, and focal positivity in few cases of in situ melanoma [102]*.*

Several studies demonstrated the ability of microRNA to regulate the expression of semaphorins or their receptors [103]. Our data support the existence of a regulatory circuitry of Sema5A expression involving miR-204, as well as c-Myb, and the anti-apoptotic protein Bcl-2 [102]*.* A more recent study also identified miR-155-5p and miR-205-5 as possible regulators of Sema5A in melanoma models. In particular, bioinformatics analysis revealed Sema5A among the target genes, but the authors furnished not further characterization of this regulation. Moreover, even if the expression of both miR-155-5p and miR-205-5 was altered in the premetastatic liver of mice carrying murine melanoma tumors, no difference in the expression of Sema5A in the premetastatic lungs and livers was evidenced between melanoma carrying mice and the control group [104]*.*

Analysis of melanoma cells derived from different areas of the same tumor demonstrated a decreased expression of Sema5A RNA transcript in BRAF mutated melanoma cells derived from the central regions of primary melanoma when compared to the BRAF negative cells derived from peripheral areas, thus indicating that modulation of Sema5A expression could be involved in divergent effects of anticancer agents [15]*.*

#### Sema6A

BRAF mutant melanoma cells, when compared to NRAS mutant ones, showed higher expression of Sema6A. Inhibition of Sema6A in BRAF mutant cells reduced chemotaxis and invasion and induced cell death. Similarly, increased overexpression of Sema6A, in NRAS mutated cells, induced anchorage-independent growth and enhanced invasion, thus, indicating Sema6A as a potential therapeutic target for melanoma treatment [105]*.*

#### Sema7A

Exposure of human melanocytes to recombinant Sema7A has been reported to induce cytoskeletal reorganization, ending in adhesion and spreading and dendrite formation [106]*.* β1 integrin mediated attachment and spreading and dendrite formation of melanocytes induced by Sema7A, while plexinC1 induced an opposed effect inhibiting Sema7A functions through inactivation of cofilin, an actin binding protein mediator of cell adhesion and migration [106]. In human melanocytes Sema7A also activated the non-receptor protein tyrosine kinase (FAK), and MAP kinase [83].

Through the interaction with β1 integrin or plexinC1 receptors, Sema7A has been also reported to contribute to the generation of a pulmonary microenvironment facilitating melanoma metastasis formation through the chitinase3-like protein1/interleukin-13 receptor α2 axis [107]*.*

### Involvement of NEUROPILINS in melanoma progression and response to therapy

Both neuropilin1 and neuropilin2 have been reported to play a pivotal role in driving the migration of neural crest cells, which give rise different derivatives, among them neurons and melanocytes, with fate specification [84, 108]*.*

One peculiarity of melanoma is the high somatic mutation frequency when compared with other type of cancers [109]. By using a melanoma somatic mutation dataset available from TCGA website, both plexins and neuropilins have been identified as essential melanoma proteins and potentially important for melanoma survival [110]*.*

#### Neuropilin1

Neuropilin1 was found to be expressed in some melanocytes, SV40T-transformed melanocytes [111–113] and in a low percentage of common and dysplastic nevi when compared to primary and metastatic melanoma [93, 111, 113–115]*.* On the contrary, a low expression of neuropilin1 in neural crest derivatives, such as melanoma has been observed by other authors [116]*.* Recently, it has been reported that neuropilin1 low expression is related to the ability of SOX10/miR-338 pathway to target its transcript in neural crest–derived cells [117]. In the same paper the authors identified neuropilin1 as a pivotal player in the resistance of melanoma cells to target therapy, showing higher expression of this protein in resistant cells. Induction of epidermal growth factor receptor by neuropilin1 through JNK kinase/SOX2 pathway was also reported to sustain cell proliferation and to mediate drug resistance. Moreover, the authors showed that cancer cells carrying a constitutively activated oncogene were not dependent on neuropilin1. In fact, contrary to what observed in carcinoma cells, in which neuropilin1 is widely expressed [118]*,* neuropilin1 knockdown did not affect either in vitro or in vivo tumor growth but, interestingly it was able to restore sensitivity to targeted therapy in drug-resistant melanoma xenografts, thus indicating neuropilin1 as a possible target to be used in combination with oncogene-targeted therapies.

Multiple oncogenic functions in melanoma for neuropilin1, together with its possible application as therapeutic target have been also reported by other authors [119]. Through multivariate Cox regression analysis, neuropilin1 has been identified as an independent prognostic marker and its expression was found associated with metalloprotease2 expression and melanoma progression, and inversely correlated with patient survival [113]. Studies with preclinical models demonstrated the ability of neuropilin1 to increase invasion and vasculogenic mimicry through inhibition of αVβ5 integrin [120], or through VEGFR-2 -dependent and -independent mechanisms [121]*.* Neurophilin1 was involved in chemotactic ability induced by placenta growth factor [120] and cooperated with placenta growth factor in promoting melanoma aggressiveness [122]*.* Moreover, the study of Rizzolio demonstrated that silencing of neuropilin1 did not affect in vivo melanoma growth [117]*.*

In the last years, several semaphorins and their receptors have been reported to show multiple roles in the immune system and to regulate cells involved in the innate or adaptive immune responses [7]. Even if this issue is out of the scope of this review, we should mentioned the paper by Hansen and Leclerc, in which in a mouse melanoma model, neuropilin1 expressed by regulatory T cells was able to drive these cells into the tumor microenvironment following VEGF release by melanoma cells. Moreover, specific ablation of neuropilin1 in T cell resulted in delayed tumor growth, thus further suggesting neuropilin1 inhibition as therapeutic approach for melanoma therapy [123]*.*

In the study of Leclerc, in vitro and in vivo experiments performed in different tumor models, including mice engrafted with mouse melanoma, demonstrated that interaction of Sema3A, a secreted protein, with its receptor neuropilin1 blocks functions of cytotoxic T lymphocytes, such as migration and tumour-specific lysis. Moreover, in vivo inhibition of neuropilin1 was able to cooperate with immunotherapy (i.e. anti-PD-1) in reducing the growth of melanoma tumors [124]*.*

These results provide a rational for the use of neuropilin1 inhibitors as therapeutic tools for melanoma, and more in general, for cancer treatment, alone or in combination with targeted or immunotherapy.

All these above reported results are in contrast with the only paper demonstrating a negative role of neuropilin1 on in vitro migratory ability of murine melanoma cells. A reduced binding to Sema3A, but not to Sema3C, was reported after silencing of neuropilin1, thus indicating the involvement of Sema3A in the effect of neuropilin1 in migratory ability of melanoma cell [125]*.*

#### Neuropilin2

Due to the absent expression in normal human melanocyte, relative lack of expression in benign melanocytic nevi, and high expression in melanoma, neuropilin2 has been suggested as a prognostic indicator in patients with melanoma [126]. Neuropilin2 has been reported to be expressed in primary and metastatic melanoma cells [45, 93, 115] and in patient-derived xenograft melanoma models developing pulmonary metastases [127]. It has been also found to correlate with malignant progression: within the group of primary melanomas, a positive correlation between neuropilin2 expression and Breslow depth has been identified [128]*.* Knockdown of neuropilin2 in metastatic melanoma cell lines inhibited in vitro cell proliferation and in vivo tumor growth and metastasis [129, 130]. As neuropilin2 is a membrane protein that can be also secreted form [131], its detection has been suggested as a possible surrogate melanoma marker to identify patients with occult metastatic melanoma [126]*.*

Mutagenesis of the soluble extracellular B domain of neuropilin2 and its overexpression in melanoma cells inhibited tumor growth, and increased the sensitivity of melanoma models to monoclonal antibodies against VEGF [132]*.* These results indicate that mutated neuropilin2 acts as a VEGF antagonist that could be used in combination with VEGF inhibitors, such as bevacizumab.

Neuropilin2 has been also identified as a useful marker in the differentiation between Spitzoid malignant melanoma and Spitz nevus [133]*.*

### Involvement of PLEXINS in melanoma progression and response to therapy

In 2009 Balakrishnan A et al. undertook a comprehensive genomic analysis of “plexinome”, as the authors defined the plexin gene family in melanoma cells*.* From this study, detecting gene copy number and variations and somatic mutations, emerged that multiple melanoma samples showed an enhancement of plexinA4 gene copy numbers. The region with plexinA4 gene amplification included BRAF. Systematic profiling of the entire plexin gene family also identified somatic mutations in highly conserved residues of plexinA4, plexinB3, and plexinC1 that were confirmed by multiple PCR amplification. Some of them (PLXNA4 p.H1736Y, PLXNB3 p.R538H) were reported to have a functional impact on the corresponding protein, thus suggesting their role in melanoma progression [134].

#### PlexinB1

PlexinB1 is expressed by melanocytes in the skin and has been reported to positively affect proliferation of melanocytes both in absence or presence of Sema4D and to contribute to their survival. Moreover, its expression is regulated by ultraviolet B, and trough the formation of a complex it modulated the activation of c-Met [95, 96]*.* The authors also indicated Sema4D/plexinB1 axis as a regulator of c-Met functions through Shp2 [96].

Concerning the effect of plexinB1 in melanoma, its role as a tumor suppressor gene has been reported by several groups, in agreement with results reported in other tumor histotypes such as breast and renal carcinoma [135, 136]*,* and contrary to results demonstrating an oncogenic function in human epithelial tumors [137]. In particular, plexinB1 expression is lost in invasive and metastatic melanoma and [138], abrogated integrin-dependent migration and activation of pp125FAK, and reduced the activity of Rho [139]*.* PlexinB1 also blocked the activation of c-Met and melanoma migration in response to HGF but, paradoxically, it activated Akt and protected from apoptosis induced by cisplatin. The authors indicated that the effect of loss of plexinB1 on melanoma progression could be due to the balance between the relative levels of c-Met suppression and Akt activation by plexinB1 [138]. Tumor promoting function by plexinB1 in c-Met independent melanoma has been also reported [138].

When overexpressed in human melanoma cells, plexinB1 inhibited in vitro growth of colonies and spheroids, and metastasis in a mouse model through its C-terminal region*.* The authors suggested inhibition of PI-3 kinase/AKT, following R-Ras-GTP hydrolysis and inactivation as a possible mechanism through which plexinB1 induced tumor suppression. Given the different response to plexinB1 modulation by primary and metastatic melanoma cells, the authors suggested that stage-depend events could overcome tumor suppressor mechanisms in late stage melanoma. Of note, the authors also identified plexinB1 as a target gene inhibited by BRAF signaling both in melanocytes and in melanoma cells, suggesting the induction of a permissive environment by BRAF/MEK axis through modulation of plexinB1 [140]*.*

Activation of PI3K pathway by both plexinB1 and plexinC1 was also identified as the cause of resistance to apoptosis induced by the two receptors [138, 141]*,* and a synergistic effect in the promotion of melanoma progression by loss of the two receptors has been suggested [138].

#### PlexinC1

Also plexinC1 is considered a tumor suppressor protein for melanoma progression. It is highly expressed in human melanocytes and is lost in primary and metastatic melanoma cells probably through a mechanism involving DNA methylation or gene deletion [83, 141]*.* Through its signalling, plexinC1 abrogated melanocyte proliferation, adhesion and migration [141]*.* Immunohistochemical analysis of specimens from primary and matched metastatic melanoma lesions and melanoma tumor microarray confirmed loss of plexinC1 in metastatic melanoma when compared with primary melanoma [106, 142]*.* An inverse correlation of plexinC1 with tumor depth of invasion has been also identified in metastatic melanoma [83, 142]*.*

LIM kinase and cofilin, important regulators of cytoskeleton dynamics and consequently cell adhesion and migration [143, 144]*,* represent downstream target of plexinC1 [142]*.*

Metastatic melanoma patients showed somatic mis-sense mutations, as well as copy number loss of plexinC1 [134]. PlexinC1 has been reported to delay in vivo growth of a mouse melanoma model only at early stages. This effect has been suggested as the consequence of negative role of plexinC1 on cell proliferation and migration probably through reduction of R-Ras activity, while activation of the pro-survival PI3-kinase Akt pathway has been proposed as responsible of lack of effect of plexinC1 at late stages of tumor growth [141]. This hypothesis contrasts with the one of Scott group evidencing similar level of plexinC1 in benign nevi and superficially invasive melanoma and indicating plexinC1 ability to promote tumor progression rather than tumor initiation [83]*.*

A positive correlation between plexinC1 and endothelin receptor B has been reported by using both transgenic mouse models, in which melanoma spontaneously develops, and in human primary melanomas. The authors also suggested the involvement of plexinC1 in the suppressive effect exerted by endothelin receptor B on melanoma [145]*.*

Regulation of plexinC1 by the long non-coding RNA CASC2 ability to sponge miR-181a and consequently to increase the expression of plexinC1 and to inhibit the proliferation and invasion of melanoma cells, has been recently reported [146]*.*

#### PlexinD1

PlexinD1 expression has been reported to positively correlate with invasive behaviour and metastatic progression, being absent in melanocytes in naevocellular naevi, dysplastic naevi, and melanoma in situ, while its level increasing with invasion level, according to Clark’s staging [88]*.* High expression of plexinD1 was found in about 90% of metastatic melanoma [88] and in cerebral melanoma metastases [147]*.*

### Clinical trials using SEMAPHORINS as targets for the therapy of cancer

A huge amount of preclinical findings raise the possibility that semaphorins/semaphorin receptors could play relevant function as biomarkers or have therapeutic potential, not only for cancer but also for other pathologies [148, 149]. Semaphorins are also reported to be predictors to cancer therapy. This is the case of class3 semaphorins [150], Sema4C [151], Sema7A [152]. Table 3 shows a list of clinical trials aimed at investigating the diagnostic efficiency of specific semaphorins and their potential role as early relapse or proliferation biomarkers in cancer. This list also includes clinical trials in which semaphorins or their receptors are investigated as possible indicators of response to cancer therapy.

**Table 3:** Clinical trialsusing Semaphorins as biomarkersfor cancer

ClinicalTrials.gov Identifier	Recruitment status	Endpoints	Cancer histotype
NCT03663153	Not yet recruiting	Semaphorin 4C as a relapse biomarker	Breast cancer
NCT03662633	Not yet recruiting	Semaphorin 4C as diagnostic value	Breast cancer
NCT02747355	Unknown	Semaphorin 3A and Neuropilin1 as proliferation biomarkers	Adreno-cortical tumors
NCT01063387 Phase 1	Unknown	Semaphorin 4C as biomarker of response to therapy	Locally advanced uterine cervical cancer exposed to extended-field irradiation
NCT03517176 Phase 1	Completed	Neuropilin1 as biomarker of response to therapy	Metastatic pancreatic cancer treated with CEND1/Paclitaxel/Gemcitabine
NCT01471470 Phase 2	Completed	Neuropilin1 as angiogenic biomarker	Unresectable advanced gastric cancer treated with Docetaxel, Capecitabine, Cisplatin, and Bevacizumab
NCT02129257 Phase 2	Completed	Semaphorins, Neuropilin1 and 2 as biomarker of response to therapy	Advanced colorectal cancer treated with combination chemotherapy with Aflibercept
NCT01478594 Phase 2	Completed	Neuropilin as biomarker of response to therapy	Metastatic colorectal cancer treated with mFOLFOX6/Tivozanib/ Bevacizumab
NCT04252456 Not Applicable	Recruiting	Neuropilin as angiogenic biomarker	Metastatic colorectal cancer treated with Folfiri/Aflibercept
NCT02854618 Not Applicable	Recruiting	Neuropilin2 asbiomarker of immune response	Metastatic breast cancer treated with Everolimus
NCT01989780 Phase 2	Unknown	Neuropilin as angiogenic biomarker	Breast cancer treated with Bevacizumab/Paclitaxel/ Endocrine therapy

Accumulating preclinical evidences indicated the use of monoclonal antibodies [153], small molecules [154], antisense oligonucleotides [155] or peptides inhibiting semaphorins/ semaphorin receptors binding [156], to modulate the expression of semaphorins or their receptors, with the goal of treating cancer or other diseases. Certain results from these studies spurred several clinical trials for cancer (Table 4).

**Table 4:** Clinical trials using semaphorins/semaphorin receptors as targets for the therapy of cancer

ClinicalTrials.gov Identifier	Recruitment status	Aim of the trial	Cancer histotype
NCT01313065 Phase 1	Completed	Safety, tolerability, pharmacokinetics and pharmacodynamics of anti-Semaphorin 4D neutralizing monoclonal antibody, VX15/2503 (Pepinemab)	Advanced solid tumors *(Patnaik A* et al *Clin Cancer Res 2016)*
NCT03268057	Completed	Safety and tolerability of VX15/2503 in combination with avelumab	Advanced non-small cell lung cancer
Phase 1, Phase 2			
NCT03690986	Recruiting	Efficacy of VX15/2503 in combination with ipilimumab or nivolumab	Stage I-IVA head and neck squamous cell cancer
Phase 1			
NCT03373188	Recruiting	Efficacy of VX15/2503 with or without ipilimumab or nivolumab	Resectable stage I-III pancreatic and stage IV colorectal cancer
Phase 1			
NCT03769155	Recruiting	Efficacy of VX15/2503 with or without ipilimumab and/or nivolumab	Resectable Stage IIIB-D Melanoma
Phase 1			
NCT03425461	Active, not recruiting	Efficacy of VX15/2503 with nivolumab or ipilimumab	Stage III or IV Melanoma
Phase I			
NCT00747734	Completed	Dose-limiting toxicities of anti-Neuropilin1 neutralizing monoclonal antibody, MNRP1685A	Locally advanced or metastatic solid tumors *(Weekes CD* et al *Invest New Drugs 2014)*
Phase 1			
NCT00954642	Completed	Efficacy of MNRP1685A in combination with bevacizumab with or without paclitaxel	Locally advanced or metastatic solid tumors *(Patnaik A* et al *CancerChemotherapyPharmacology 2014)*
Phase 1			

Monoclonal antibodies constitute the first and unique approach inhibiting semaphorins or their receptors that reached clinical studies: in 2015 the VX15/2503 (Pepinemab) humanized IgG4 neutralizing antibody having high affinity for rodent, primate and human Sema4D has been identified. Then the pharmacokinetics, toxicology and pharmacodynamics of VX15/2503in rats and macaque shave been reported [157, 158]. These findings together with the evidence that monoclonal antibodies against Sema4D improved the response to immune checkpoint inhibitors [159] indicated the monoclonal antibody as a potential therapeutic strategy for subjects with cancer and supported the continued investigation of VX15/2503 in clinical studies. Table 4 shows a list of clinical studies performed to evaluate safety and efficacy of VX15/2503 in cancer. In particular, a first-in-human study performed in patients with advanced solid tumors demonstrated that VX15/2503 was well tolerated and indicated an immune-mediated mechanism of action (NCT01313065) [160]. Preliminary results of a phase 1b/2 study act to evaluate safety and efficacy of VX15/2503 in combination with Avelumab (anti-PD-L1) in non-small cell lung cancer have been presented in 2020 at the annual meeting of the American Society of Clinical Oncology (NCT03268057). The authors indicated the ability of VX15/2503 to increase the frequency of objective responses and to extend progression-free survival relative to single agent avelumab (https://meetinglibrary.asco.org/record/183040/abstract). Four phase I trial studies are recruiting patients with head and neck cancer (NCT03690986), resectable pancreatic and colorectal cancer (NCT03373188), as well as advanced melanoma (NCT03425461, NCT03769155) to analyse the efficacy of VX15/2503 in combination with the immune checkpoint inhibitors ipilimumab (anti-CTLA4) and nivolumab (anti-PD1). In particular, the first study of melanoma is carried out in melanoma patients who have progressed on anti-PD1/L1 based checkpoint inhibitors and the objectives are to determine i) safety and tolerability of the combination of VX15/2503 with nivolumab, or ipilimumab; ii) recommended phase II dose and schedule of the combination of VX15/2503 with nivolumab, or ipilimumab; iii) adverse event profile for the agent combinations; iv) clinical response of patients treated with maximum tolerated dose or maximum administered dose of the combination; v) whether adding VX15/2503 to PD1 or CTLA-4 blockade can increase T-cell infiltration into tumors and whether change in T-cell infiltration is associated with response (NCT03425461). The second study on melanoma is carried out in patients with resectable metastatic melanoma and the objectives are those for the first study and also include immune profile of involved and uninvolved lymph nodes and peripheral blood (NCT03769155).

The monoclonal antibodies approach has been also used to evaluate the safety/tolerability and efficacy of semaphorin receptor inhibitors in cancer patients, but the outcome has been disappointing (Table 4). In fact, even if the fully human anti-neuropilin1 IgG1 antibody MNRP1685A (Vesencumab) was generally well-tolerated (NCT00747734) [161], unfortunately side effects, including thrombocytopenia, were observed in a phase Ib study in combination with bevacizumab and paclitaxel in patients with advanced solid tumors and did not support its further testing (NCT00954642) [162]. To overcome the limitation of low penetration in solid tumors of these large size monoclonal antibodies, nanobodies targeting neuropilin1 have been developed and found to enhance survival of tumor-carrying mice [163].

Small molecules also represent a possible approach to inhibit the expression/activity of semaphorins/semaphorin receptors axis. Unlikely, at present only preclinical findings support the use of small molecules inhibiting Sema3A (SM-216289, Xanthofulvin; SM-345431, Vinaxanthone) for the treatment of disease not including cancer [154, 164–166]. These findings could support the use of small molecules for human patients with tumor histotypes in which Sema3A exert a pro-tumorigenic effect, such as colorectal carcinoma [163] or nasopharyngeal carcinoma [167].

As indicated by preclinical studies [155, 168] also antisense oligonucleotides could constitute a possible strategy to be exploited for semaphorins downregulation, but at present there are no clinical studies evaluating this possibility.

Considering the tumor suppressive effect of some semaphorins, recombinant proteins could also represent a possible strategy to treat cancer. However, as of today, there is only preclinical published evidence supporting that semaphorins with suppressive functions may be a therapeutic target in cancer. This is the case of the mutated, uncleavable variant of Sema3E that was validated as a tumor/angiogenesis suppressor following its delivery in tumor or choroidal neovascularization models [169, 170]. Full-length Sema3C inhibited of tumor lymphangiogenesis and metastasis of breast carcinoma model [171]. More recently, also the superagonist Sema3A mutant has been reported to be an effective in vivo vascular normalizing agent [172]*.*

## Discussion

This paper reviews the crosstalk between hypoxia and semaphorins or semaphorin receptors in cancer models with particular attention to melanoma.

Due to their important function in the signal transduction, semaphorins represent crucial regulatory factors in the crosstalk between cancer cells and tumor microenvironment. Together with their cognate receptors, they are considered one of major pivotal player of cell-cell communication and regulators of tumor progression and response to therapy, as well as cellular metabolism and genomic stability. In many cases, deregulated activity of semaphorins, plexins or neuropilins can modify the normal tissue homeostasis, through the release of inhibitory signals or through the activation of transformation events. Many HRE, have been found in the promoter of different semaphorins and their receptors, for this reason these molecules are reported to mediate the response to hypoxia of tumor or stromal cells.

A wide subset of semaphorins and their receptors play a crucial role in melanoma pathobiology and response to therapy: some of them being able to promote melanoma angiogenesis and metastatization (Sema5A, Sema6A, Sema7A, Neuropilin1 and 2, PlexinD1), while other being involved in the inhibition of tumor development or progression (Sema3A, Sema3B, Sema3F, Sema4D, PlexinB1, PlexinC1). Contrasting results have been evidenced regarding Sema3E, showing either positive or negative effect in melanoma progression.

Several studies also suggested the use of particular semaphorins as diagnostic [107] or prognostic [86] biomarkers for melanoma.

In spite of some spectacular results for melanoma therapy, thanks to the advent of targeted therapy and immunotherapy, these treatments are often associated with the emergence of drug resistance. Thus, identifying novel approaches to treat melanoma and to reduce its pathogenicity is an urgent need. In this context some semaphorins associated with melanoma progression could represent possible targets against which new therapies could be directed. Findings from preclinical studies spurred several clinical trials aimed at investigating the diagnostic efficiency or the potential role as a relapse or proliferation biomarker or indicator of response to cancer therapy of selected semaphorins/receptors, mostly represented by Sema3A, Sema4C and neuropilin1.

A huge amount of preclinical studies also raise the possibility of semaphorins/semaphorin receptors having therapeutic potential for cancer treatment. After the demonstration of safety of VX15/2503, an anti-Sema4D antibody, in patients with advanced solid tumors [160], several clinical studies are recruiting cancer patients to analyse the efficacy of this antibody in combination with immune checkpoint inhibitors. In particular, two pilot phase 1 clinical trials are recruiting patients with advanced melanoma stage, to study the efficacy of VX15/2503 in combination with nivolumab or ipilimumab which, together with targeted therapy, represent the standard care of melanoma. Of note, the evidence that Sema4D, together with Sema6A, is the most expressed protein in melanoma, being expressed in 100% of melanoma tissues **(**Fig. 4**b)**. Moreover, given the relevance of Sema4D both in cancer and stromal cells, VX15/2503 could act by interfering with the crosstalk between tumor cells and cells belonging to the tumor microenvironment. In a short period of time we hope to have positive evidence about the response of cancer patients to VX15/2503 in combination with immune checkpoint inhibitors.

Another monoclonal antibody that reached clinical studies is MNRP1685A, an anti-neuropilin1 antibody, which showed a disappointing outcome, probably for its large size. Further studies are necessary to investigate the relevance of nanobodies against human neuropilin1 in overcoming the limitation observed in the first clinical trials. The advantage of antibodies or other molecules capable of inhibiting neuropilin1 is due to the fact that this receptor, in addition to binding different semaphorins, also shows semaphorin-independent function, binding proangiogenic factors or being essential for the functionality of cells belonging to the tumor microenvironment. On the other hand, the multifaceted role of this receptor in different cells could bring to undesired side effects.

Even if the possibility to use large recombinant proteins or antisense oligonucleotides for clinical application still represents a big challenge, we hope that in a near future preclinical findings could provide a rationale for the further development of these approaches, as well as antibodies and small molecules, to target human semaphorins or their receptors and to bring them from the bench to the bedside.

Further studies are also required to use semaphorins showing tumor suppressor activity as a possible strategy to treat cancer: preclinical evidence, mostly performed on Sema3 class, strongly encourages this possibility.

Considering the ability of several semaphorins to regulate signalling pathways associated with stem-cell like phenotype, blocking these semaphorins could also impact on cancer stem cells self-renewal or other abilities, thus reducing cancer stemness and consequently hampering tumor progression.

In conclusion, many studies are focused, and are still needed, to understand the precise function and signalling of semaphorins, and to in-depth investigate the possibility to target their activities in melanoma, and more in general, in cancer or other diseases.

## Abbreviations

HIF-1: Hypoxia-inducible factor-1.
HRE: Hypoxia response elements.
PDZ: PSD95/Dlg/ZO-1.
VEGF: Vascular-Endothelial Growth Factor.
Sema3A: Semaphorin3A.
Sema3B: Semaphorin3B.
Sema3C: Semaphorin3C.
Sema3E: Semaphorin3E.
Sema3F: Semaphorin3F.
AKT: Protein kinase B.
Sema4D: Semaphorin4D.
TNM: Tumor-Node-Metastasis.
ADAM17: ADAM metallopeptidase domain 17.
VEGFR-2: VEGF receptor-2.
circHIPK3: circular HIPK3.
ERK: Extracellular-signal-Regulated Kinase.
GEPIA: Gene Expression Profiling Interactive Analysis.
TCGA: The Cancer Genome Atlas Program.
Sema4F: Semaphorin4F.
Sema4G: Semaphorin4G.
Sema5A: Semaphorin5A.
Sema6A: Semaphorin6A.
Sema6B: Semaphorin6B.
Sema6C: Semaphorin6C.
Sema6D: Semaphorin6D.
Sema7A: Semaphorin7A.
c-Met: tyrosine-protein kinase Met.
Shp2: Src homology region 2 domain-containing phosphatase-2.
CRISP/Cas: Clustered Regularly Interspaced Short Palindromic Repeats- associated protein.
BRAF: B-Raf Proto-Oncogene, Serine/Threonine Kinase.
NRAS: Neuroblastoma Ras viral oncogene homolog.
FAK: Focal Adhesion Kinase.
SOX10: SRY-related HMG-box 10.
JNK kinase: c-Jun N-terminal kinase.
MEK: MAPK/ERK Kinase.
CASC2: Cancer Susceptibility Candidate 2.
